# A review of the application of autologous blood transfusion

**DOI:** 10.1590/1414-431X20165493

**Published:** 2016-08-01

**Authors:** J. Zhou

**Affiliations:** Department of Blood Transfusion, Military General Hospital of Beijing, Beijing, China

**Keywords:** Autologous blood transfusion, Preoperative autologous blood donation, Acute normovolemic hemodilution, Intraoperative and postoperative autotransfusion

## Abstract

Autologous blood transfusion (ABT) has been gradually attracting more attention due to the increasingly prominent problem of blood transfusion safety and blood shortage in recent years. With the rapid development of blood conservation techniques, blood component separation technology, blood transfusion medicine and a constant increase in clinical needs, ABT technology has been expanded and innovated to a large degree. In this study, the development of preoperative autologous blood donation (PABD), acute normovolemic hemodilution (ANH), intraoperative and postoperative autotransfusion, and other new technologies and theories are reviewed and existing questions are analyzed. Challenges and applications are also discussed in order to provide reference for peers.

## Introduction

With the improvement of medical technology and the growing discrepancy between blood supply and demand, autologous blood transfusion (ABT) has attracted more attention in recent years; its safety and effectiveness has gradually become a subject of interest. Shortage in blood supply has greatly promoted the improvement of blood preservation and safety, but some risks remain in blood transfusion. Blood transfusion has both favorable and unfavorable consequences: it can save a life but can also be fatal. Allogeneic blood transfusion not only causes adverse reactions that would lead to the spread of various infectious diseases, but also increases the economic and psychological burden of patients and their families. ABT can avoid the serious harm caused by allogeneic blood transfusion (1-4), alleviate blood shortage and save blood resources, while lightening the burden of patients. Therefore, ABT has gained more attention, has become a common demand in clinical practice (5), and is becoming widely used clinically.

Allogeneic blood transfusion is a potentially hazardous method similar to allogeneic organ transplantation. It may cause long-term effects on immunization, which would likely lead to micro-thrombosis, blood coagulation and hemolytic reactions. Since 1980, with advantages such as the prevention of the spread of diseases, less adverse reactions, saving of blood resources, high safety and efficiency, ABT has been widely recognized by clinical staffs and has been widely used in treatments for anemia and surgical bleeding. In recent years, with the increased knowledge of medical staffs, blood management personnel, patients and their families, ABT has been gradually accepted and the volume of autologous blood storage in hospitals has also gradually increased. ABT can avoid the spread of blood-borne diseases and immunosuppression, and sometimes it is the only blood supply in cases where the needed type of blood is not available in a short time. ABT includes three options: preoperative autologous blood donation (PABD), acute normovolemic hemodilution (ANH), and intraoperative and postoperative autotransfusion.

## Preoperative autologous blood donation (PABD)

PABD refers to a technique in which the patient's own blood is collected and saved for a period of time before the surgery, and reinfused back during the surgery. Repeated blood donations before surgery can stimulate bone marrow cell proliferation, stimulate erythrocyte regeneration, increase hematopoietic function in patients after surgery, accelerate the patient’s hematopoietic recovery after surgery, is conducive for wound healing, and reduces the chances of infection caused by immunoreaction from allogeneic blood transfusion (6,7). These are the unique advantages of PABD, compared with other methods. Thus, it has been widely used in clinical treatment and in the surgical field (8-10), achieving remarkable results. However, in clinical practice, storage of autologous blood before surgery has some limitations. For example, predeposited autotransfusion is mainly applied to young patients and not old patients. Its applications include lumbar disc herniation, scoliosis, hip surgery and surgical hematorrhea in pregnant women. Furthermore, it has a favorable effect in reducing the adverse reactions of blood transfusion, maintaining normal blood indexes, improving abnormal blood rheology, and alleviating the lack of blood supply (11-16).

In addition to traditional transfusion patterns, preoperative ABT has been improving as follows: 1) Reasonably basic treatments are performed according to the patient's specific situation, which best prepares the body for blood donation; 2) An appropriate amount of crystalloid liquid is transfused into the body before and after the blood donation to slightly dilute the blood, reduce blood viscosity, improve microcirculation, and reduce blood damage and actual blood loss during surgery. The mild hemodilution does not have a high negative impact on the oxygen-carrying capacity of blood, blood coagulation or in the heart, brain and kidneys, while allowing the collection of enough autologous blood; 3) When a large amount of blood needs to be collected, erythropoietin and iron are administered to promote the formation and maturation of erythrocytes in patients, preventing preoperative anemia. Studies have shown that the combined application of erythropoietin and iron can promote hematopoiesis in a short time (17). The preoperative administration of erythropoietin can increase hemoglobin levels in patients, reduce the amount of autologous and allogeneic blood transfusion, and also contribute to the recovery of patients (18). Therefore, the improved ABT has a number of advantages such as the mild dilution of blood, reduction in blood viscosity, improved microcirculation and prevention of hypoxia caused by anemia after blood donation. This optimized PABD (adjustment of physical condition by basic treatment, supplement of crystalloid liquid, and intraoperative administration of erythropoietin) is a safe and effective preoperative blood preparation program for patients who are scheduled to undergo elective cardiac surgery. The technique is successful in cases in which no adverse reaction occurs during the blood transfusion process, hemoglobin and hematocrit levels are within the normal range before and after autologous blood donation, before and after transfusion, and before discharge. Therefore, the number of days of preoperative hospitalization, in the ICU, of postoperative hospitalization and of total hospitalization are significantly reduced, as well as the incidence of postoperative complications in heart surgery (19).

## Acute normovolemic hemodilution ABT

Acute normovolemic hemodilution (ANH) is a method of perioperative ABT first used in 1946 and now widely used in clinical practice (20-22). ANH is generally performed after anesthesia and before the beginning of the main steps of surgery. A predetermined amount of autologous blood is rapidly withdrawn and stored in the operating room, while an equivalent volume of crystalloid or colloidal liquids is transfused into the body of patients in order to appropriately dilute the blood, reduce the hematocrit, and reduce the loss of visible components of blood during surgery. Then, the collected autologous blood is transfused back into the patient when transfusion indications appear or before the end of the surgery (20,21,23). ANH autologous transfusion can effectively reduce intraoperative erythrocyte loss. It has been widely used in postpartum hemorrhage, and cancer and orthopedic surgeries, such as joint replacement and spine surgery. These surgeries often have large traumatic areas and a large amount of bleeding, and patients often need blood transfusions (24-26). ANH advantages include the following. 1) Due to the supplement of crystalloid or colloidal liquids, ANH dilutes the blood during surgery, reducing the concentration of erythrocytes in blood circulation during surgery. In turn, this reduces erythrocyte loss and improves the body's tolerance, reducing actual blood loss during surgery (23,27). 2) ANH is the only method that provides fresh autologous blood, in which the function of platelet and clotting factors are rarely affected and few red blood cells are lost. 3) Compared with preoperative autologous transfusion, ANH autotransfusion is a simple operation, has low cost, short blood storage time and low visible component damage, and prevents repeated blood withdrawal. For some patients for which autologous blood storage is not suitable, such as patients with suspected bacteremia, ANH can be safely performed under the intensive care of an anesthesiologist. Also, cancer surgery is not suitable for blood recycling, but ANH can be performed (23). ANH ABT is particularly suitable for patients with Rh D negative traits, irregular antibodies, difficult ABO blood typing, or with other blood matching problems. Combined with autotransfusion, ANH effectively prevents postoperative bleeding and anemia, provides a safe and effective autologous transfusion method, and therefore should be available for general use.

Studies have revealed that patients with preoperative Hb >110 g/L, Hct >33%, PLT >100×10^9^/L, normal prothrombin time and normal cardio-pulmonary function could undergo ANH ABT (28). ANH ABT has been successfully used in prolonging the survival of lung cancer patients (29), dealing with hemorrhage in orthopedic surgery and postpartum hemorrhage in the Rh (D) negative patients, and providing a safe and effective mode of blood transfusion (25,30). The application of ANH ABT in malignant tumor surgery can reduce the amount of allogeneic blood transfusion, save money and prevent blood borne diseases. At the same time, it can induce the production of a variety of immune factors (25) without affecting liver and blood coagulation functions (26).

## Intraoperative or postoperative autotransfusion

Intraoperative or postoperative autotransfusion refers to a method of transfusion in which blood in the body cavity of a patient, blood lost during surgery and postoperatively drained blood, can be recovered through a blood recovery device. Then, blood undergoes anticoagulation, filtration and washing, and is finally transfused back to the patient (31). The American Association of Blood Banks guidelines recommend that intraoperative or postoperative autotransfusion should be performed in surgeries where a large amount of bleeding (more than 20% total volume) is anticipated (32). However, it has been performed in surgeries with a relatively small amount of bleeding (400 mL, for example) (33). It remains unclear whether patients would benefit from the application of intraoperative or postoperative autotransfusion in certain circumstances.

At present, the application of intraoperative or postoperative autotransfusion has been expanded. Its application in surgeries with a small amount of bleeding in healthy adults can moderately improve early postoperative Hb levels and tissue oxygenation, but has no significant effect on postoperative recovery (34). Patients who underwent autotransfusion had a lighter suppression of cellular immune function, and recovered their cellular immune function faster compared with allogenic blood transfusion (35). Intraoperative or postoperative autotransfusion can also be combined with preoperative ABT in elective major surgeries to improve the effect of blood transfusion and the prognosis of patients (36). Intraoperative or postoperative autotransfusion applications in cancer surgery remain controversial, since some clinicians fear that it may cause cancer cell proliferation and metastasis (37,38). However, other studies have revealed that it can be safely applied to certain tumor surgeries (39), with the required specific assessments before and during surgery such as tumor metastasis and recurrence, and the use of a leukocyte filter (40-42).

## Development trends and challenges of ABT

Currently, traditional approaches of autologous whole blood transfusion such as leapfrog and step-by-step accumulation approaches continue to be applied at most hospitals. However, traditional approaches of preoperative blood storage also have many shortcomings: large blood volume, low concentration of blood components, especially platelets, and high adverse reaction rate. Blood conservation methods are not ideal; due to storage of whole blood at 4°C, a large amount of active components is lost or their activities are lost (43). In addition, the blood volume of a single donation should not exceed 500 mL and 12% of the total blood volume, which leads to repeated autologous blood donations or the application of allogenic blood in patients who require a large amount of blood in elective surgery. Furthermore, traditional autologous transfusions require blood donation to be conducted at least once a week before surgery. The "leapfrog" method requires donations at least one month in advance, and the step-by-step accumulation method requires blood collection 20 days in advance. These methods and concepts are contrary to the requirements of hospitals in terms of the average number of hospitalization days, preoperative preparation days and other indexes, restricting the implementation and popularization of ABT (44). Thus, ABT techniques and concepts urgently need to be further explored and researched, especially in blood collection methods and timing, in order to highlight the role of ABT in clinical transfusion.

Blood cell separation technology began in the 1980s, and has been used in clinic for many years mainly for hematopoietic stem cell collection, granulocyte collection, plasmapheresis, blood component removal and other therapeutic uses (45,46). This technology is widely used for allogeneic blood component collection such as platelets, plasma and erythrocytes (47). In preoperative autologous transfusion, this technique can be used to collect red cell and platelet concentrate, and plasma, according to the patient's requirements. These components are separately stored according to their respective preservation requirements in order to maintain their physiological activity (48). At the same time, since the liquid volume that flows in and out the body is kept in balance, this technology can ensure the safety of blood components collection. In addition, only concentrated blood components are collected; thus, the blood volume in patients before and after the collection does not significantly change. Therefore, this technology has a secure and efficient advantage when applied in preoperative autologous transfusions.

Using a blood apheresis apparatus, 2 units or more of red blood cells can be collected in one time. In a comparative study, 2 units of red blood cells were collected in two ways: whole blood collection and apheresis collection. Results revealed that in the apheresis collection, only the concentrated blood components were collected and the blood volume of the patient did not significantly change before and after the collection, thereby ensuring safety (48,49). Compared with whole blood collection, patients who underwent apheresis had fewer hospitalization days, faster recovery of postoperative hemoglobin levels, and lower incidence of adverse reactions. However, at present, the collection of more than 2 units of autologous erythrocyte or other blood components using a blood apheresis apparatus has not been reported. Some scholars believe that in preoperative autologous transfusion, apheresis collection of larger amounts or multi-component collection is a very promising pattern that could replace whole blood collection.

Using existing techniques, the preservation time of platelets is short. This is another reason for restricting the development of preoperative ABT. Platelets collected using tubes made in China can be stored for 1–3 days, while storage using imported tubes lasts 5–7 days. Therefore, it is necessary to improve platelet storage technology to extend autologous platelet storage time. Platelet cryopreservation technology has continuously gained more attention worldwide. In order to avoid damaging the platelet membrane during cryopreservation, which affects platelet function after rewarming, a cryoprotectant must be added. Dimethyl sulfoxide (DMSO) is a cryoprotectant, as well as an enhancer of cell fusion and permeability (50,51). The application of DMSO in the cryopreservation of apheresis platelets has been included in the 1998 European "Blood Component Preparation, Application and Quality Assurance Guidelines". China's Food and Drug Administration also indicates that DMSO can be used for the cryopreservation of platelets and stem cells, and for clinical infusion without washing. A study revealed that when DMSO with a final concentration of 6% was added, platelets in long-term storage at -80°C kept a considerable quality and stable function (52). Therefore, in the future, this strategy can be used clinically. This would extend platelet life, improve the hemostatic function, and ensure that the patient's autologous platelets maintain their physiological function.

The existing ABT technology cannot completely satisfy the clinical requirements of transfusion. Hence, there is an urgent need for new technology and new concepts. We hope that the blood apheresis technology and platelet preservation technique can be applied in ABT, in the near future. In upcoming studies, we plan to determine blood collection programs according to patient's conditions and surgical risk, in order to evaluate the effect of different collection amounts and times on the recovery of patients. For the introduction of blood apheresis, including red blood cell, plasma, platelet and peripheral blood stem cell PABDs, a safe and efficient preoperative autologous transfusion technology needs to be established. Preoperative autologous blood apheresis transfusion would help increase autologous transfusion rate, further reduce allogeneic blood transfusion, and its associated risks. Therefore, as an ideal transfusion technology, ABT would become one of the future directions of blood transfusion development.

In summary, compared with allogeneic blood transfusion, ABT has irreplaceable advantages such as avoiding allergies, immunosuppression, hemocytolysis and other adverse reactions. It is a safe, effective and affordable method of blood transfusion, which has broad prospects of clinical application. Especially for patients with rare blood types, patients who have been transfused with allogeneic blood and produced irregular antibodies, and patients with other blood matching problems, this technology is of great significance (53), and provides convenience for elective surgeries. The most prominent clinical features of ABT is that it can instantly provide patients with fresh blood that has identical type, quickly replenish the patient's blood volume, and improve oxygen carrying capacity (54,55), maintaining an effective blood circulation. In addition, autologous blood has low acid content and normal K^+^ concentration, relatively higher 2,3-diphosphoglycerate levels and provides better cell vitality, preventing complications such as hyperkalemia. However, due to its limitations, we should gradually develop more reasonable approaches of autotransfusion to adapt the technique for clinical treatment and surgery. Autologous whole blood transfusions should be gradually replaced by autologous apheresis transfusions, making clinical autotransfusion safer, cost-effective, and more acceptable to patients and their families.

## References

[1] Chalfin HJ, Frank SM, Feng Z, Trock BJ, Drake CG, Partin AW (2014). Allogeneic versus autologous blood transfusion and survival after radical prostatectomy. Transfusion.

[B02] Tesic I, Sekulic J, Arbutinov V, Popov D, Velisavljev D (2014). Autologous blood transfusion in patients undergoing hip replacement surgery. Med Pregl.

[B03] Dietrich W, Thuermel K, Heyde S, Busley R, Berger K (2005). Autologous blood donation in cardiac surgery: reduction of allogeneic blood transfusion and cost-effectiveness. J Cardiothorac Vasc Anesth.

[4] Karger R, Weber C, Schmidt J, Kretschmer V (2007). Characterization of immune system alterations following preoperative autologous blood donation for elective hip replacement surgery. Transfus Med.

[5] Tsuno NH, Nagura Y, Kawabata M, Matsuhashi M, Sone S, Ikeda T (2013). The current status of autologous blood transfusion in Japan - the importance of pre-deposit autologous blood donation program and the needs to achieve patient blood management. Transfus Apher Sci.

[6] Xu YA, Zhang J, Zhou AG (2009). Clinical study of predeposit autologous transfusion in total knee replacanent. J Traumatic Surg.

[7] Jakovina Blazekovic S, Bicanic G, Hrabac P, Tripkovic B, Delimar D (2014). Pre-operative autologous blood donation versus no blood donation in total knee arthroplasty: a prospective randomised trial. Int Orthop.

[8] Long MY, Liu ZH, Zhu JG (2014). Comparative analysis of autologous blood transfusion and allogeneic blood transfusion in surgical patients. Int J Clin Exp Med.

[B09] Martin K, Keller E, Gertler R, Tassani P, Wiesner G (2010). Efficiency and safety of preoperative autologous blood donation in cardiac surgery: a matched-pair analysis in 432 patients. Eur J Cardiothorac Surg.

[10] Kapadia BH, Banerjee S, Issa K, McElroy MJ, Harwin SF, Mont MA (2013). Preoperative blood management strategies for total knee arthroplasty. J Knee Surg.

[11] Singbartl G (2011). Pre-operative autologous blood donation: clinical parameters and efficacy. Blood Transfus.

[B12] Zheng Y, Kong WB, Sun YH (2013). Preoperative autologous blood donation in patients with elective surgery and blood protection. Chinese J Blood Transf.

[B13] Solves P, Carpio N, Moscardo F, Bas T, Canigral C, Salazar C (2013). Results of a preoperative autologous blood donation program for patients undergoing elective major spine surgery. Transfus Apher Sci.

[B14] Tamai K, Terai H, Toyoda H, Suzuki A, Yasuda H, Dozono S (2015). Which is the best schedule of autologous blood storage for preoperative adolescent idiopathic scoliosis patients?. Scoliosis.

[B15] Yamamoto Y, Yamashita T, Tsuno NH, Nagamatsu T, Okochi N, Hyodo H (2014). Safety and efficacy of preoperative autologous blood donation for high-risk pregnant women: experience of a large university hospital in Japan. J Obstet Gynaecol Res.

[16] Kennedy C, Leonard M, Devitt A, Girardi FP, Cammisa FP (2011). Efficacy of preoperative autologous blood donation for elective posterior lumbar spinal surgery. Spine.

[17] Tan W (2007). Application of autologous blood transfusion in cardiosurgery with extracorporeal circulation. Med Recapitulate.

[18] Vargas-Pabon M, Diaz-Trapiella A, Hurtado MJ, Diaz VN, Cerra Sabio JL (2005). Erythropoietin as adjuvant to pre-operative autologous blood donation in total hip arthroplasty: new algorithm for use. Transfus Apher Sci.

[19] Dai P, Che C, Zhang WM, Zhu HY, Yang F (2014). Evaluation of the optimized schemes for preoperative autologous donation in cardiac elective surgery. Chinese J Blood Transfus.

[20] Huang SS, Zhou XH (2012). Diluted autotransfusion combined with controlled hypotension on patients with orthopedics of liver fuction and blood coagulation fuction of operation. J Clin Hematol.

[B21] Oppitz PP, Stefani MA (2013). Acute normovolemic hemodilution is safe in neurosurgery. World Neurosurg.

[22] Jones SB, Whitten CW, Monk TG (2004). Influence of crystalloid and colloid replacement solutions on hemodynamic variables during acute normovolemic hemodilution. J Clin Anesth.

[23] (1996). Practice Guidelines for blood component therapy: A report by the American Society of Anesthesiologists Task Force on blood component therapy. Anesthesiology.

[24] Goldberg J, Paugh TA, Dickinson TA, Fuller J, Paone G, Theurer PF (2015). Greater volume of acute normovolemic hemodilution may aid in reducing blood transfusions after cardiac surgery. Ann Thorac Surg.

[B25] Yoko I, Atsushi K, Akina T, Sugiyama K (2012). Preoperative autologous blood donation and acute normovolemic hemodilution affect intraoperative blood loss during sagittal split ramus osteotomy. Transfus Apheresis Sci.

[26] Guo JR, Jin XJ, Yu J, Xu F, Zhang YW, Shen HC (2013). Acute normovolemic hemodilution effects on perioperative coagulation in elderly patients undergoing hepatic carcinectomy. Asian Pac J Cancer Prev.

[27] Ickx BE, Rigolet M, Van Der Linden PJ (2000). Cardiovascular and metabolic response to acute normovolemic anemia. Effects of anesthesia. Anesthesiology.

[28] Hill J, James V (2003). Survey of autologous blood transfusion activity in England (2001). Transfus Med.

[29] Dong L, Liu SW, Guan JY (2014). The effect of acute normovolemic hemodilution autologous transfusion on lung cancer. J Clin Hematol.

[30] Cao JW, Yao ZJ, Liao YQ (2014). The study of the security of acute normovolemic hemodilution autologous transfusion in orthopedic perioperation. J Clin Trasfus Lab Med.

[31] Ashworth A, Klein AA (2010). Cell salvage as part of a blood conservation strategy in anaesthesia. Br J Anaesth.

[32] Committee AAT (1997). Guidelines for blood recovery and reinfusion in surgery and trauma.

[33] Esper SA, Waters JH (2011). Intra-operative cell salvage: a fresh look at the indications and contraindications. Blood Transfus.

[34] Li HG, Zhang ZY, Huamg YG, Yu XR (2015). Effectiveness of intraoperative autotransfusion in mild-bleeding surgery: a randomized controlled trial. Med J Peking Union Med College Hosp.

[35] Wei M, Liu J, Tu L, Liang YH, Xi YJ, Zhang YH (2014). Effect of autologous blood transfusion on cytoimmunity function in patients undergoing cardiac operation. Chinese J Blood Transfus.

[36] Walsh TS, Palmer J, Watson D, Biggin K, Seretny M, Davidson H (2012). Multicentre cohort study of red blood cell use for revision hip arthroplasty and factors associated with greater risk of allogeneic blood transfusion. Br J Anaesth.

[37] Foltys D, Zimmermann T, Heise M, Kaths M, Lautem A, Wisser G (2011). Liver transplantation for hepatocellular carcinoma - is there a risk of recurrence caused by intraoperative blood salvage autotransfusion?. Eur Surg Res.

[38] Futamura N, Nakanishi H, Hirose H, Nakamura S, Tatematsu M (2005). The effect of storage on the survival of cancer cells in blood and efficient elimination of contaminating cancer cells by a leukocyte depletion filter. Am Surg.

[39] Liumbruno GM, Bennardello F, Lattanzio A, Piccoli P, Rossetti G (2011). Recommendations for the transfusion management of patients in the peri-operative period. II. The intra-operative period. Blood Transfus.

[40] Tomimaru Y, Eguchi H, Wada H, Hama N, Kawamoto K, Kobayashi S (2013). Predicting the necessity of autologous blood collection and storage before surgery for hepatocellular carcinoma. J Surg Oncol.

[B41] Zhu SY, Tang YM, Zhang HG, Su X (2015). Research advances in the application of intraoperative, salvaged and autologous blood transfusion in major oncologic surgery. Chinese J Blood Transfus.

[42] Singbartl G, Schreiber J, Singbartl K (2009). Preoperative autologous blood donation versus intraoperative blood salvage: intraindividual analyses and modeling of efficacy in 1103 patients. Transfusion.

[43] Vanderlinde ES, Heal JM, Blumberg N (2002). Autologous transfusion. BMJ.

[44] Chen L, Zhou XD (2013). The clinical research progress of autologous blood transfusion. Internal Med China.

[45] Safwat AM, Bush R, Prevec W, Reitan JA (2002). Intraoperative use of platelet-plasmapheresis in vascular surgery. J Clin Anesth.

[46] Carter CA, Jolly DG, Worden CE, Hendren DG, Kane CJ (2003). Platelet-rich plasma gel promotes differentiation and regeneration during equine wound healing. Exp Mol Pathol.

[47] Li H, Chen YY, Feng LH (2015). Red blood apheresis of COM.TEC blood cell separators for therapentic clincal observation. Chinese J Clin.

[48] Li H, Jia F, Shi HM, Wang Y, Wang DQ (2013). Apheresis application of predeposit auotransfusion in surgical operation. Chinese J Med.

[49] Sun GX, Xi ZY, Li H, Shi HM, Wng DQ (2015). Transfusion of pre-stored apheresis autologous red blood cell in retroperitoneal tumor elective surgery. Labeled Immunoassays Clin Med.

[50] Slichter SJ, Jones M, Ransom J, Gettinger I, Jones MK, Christoffel T (2014). Review of *in vivo* studies of dimethyl sulfoxide cryopreserved platelets. Transfus Med Rev.

[51] Valeri CR, Giorgio GR (2015). Response to the article on dimethyl sulfoxide-cryopreserved platelets published in Transfusion Medicine Reviews Volume 28 (4). Transfus Med Rev.

[52] Yilmaz S, Cetinkaya RA, Eker I, Unlu A, Uyanik M, Tapan S (2016). Freezing of apheresis platelet concentrates in 6% dimethyl sulfoxide: the first preliminary study in Turkey. Turk J Haematol.

[53] Brown CV, Foulkrod KH, Sadler HT, Richards EK, Biggan DP, Czysz C (2010). Autologous blood transfusion during emergency trauma operations. Arch Surg.

[54] Kumar N, Chen Y, Nath C, Liu EH (2012). What is the role of autologous blood transfusion in major spine surgery?. Am J Orthop.

[55] Mason L, Fitzgerald C, Powell-Tuck J, Rice R (2011). Intraoperative cell salvage versus postoperative autologous blood transfusion in hip arthroplasty: a retrospective service evaluation. Ann R Coll Surg Engl.

